# Opioid Dose Trajectories and Associations With Mortality, Opioid Use Disorder, Continued Opioid Therapy, and Health Plan Disenrollment

**DOI:** 10.1001/jamanetworkopen.2022.34671

**Published:** 2022-10-05

**Authors:** Ingrid A. Binswanger, Susan M. Shetterly, Stanley Xu, Komal J. Narwaney, David L. McClure, Deborah J. Rinehart, Anh P. Nguyen, Jason M. Glanz

**Affiliations:** 1 Institute for Health Research, Kaiser Permanente Colorado, Aurora; 2 Chemical Dependency Treatment Services, Colorado Permanente Medical Group, Aurora; 3 Division of General Internal Medicine, Department of Medicine, University of Colorado School of Medicine, Aurora; 4 Department of Health Systems Science, Kaiser Permanente Bernard J. Tyson School of Medicine, Pasadena, California; 5 Department of Research and Evaluation, Kaiser Permanente Southern California, Pasadena; 6 Center for Clinical Epidemiology and Population Health, Marshfield Clinic Research Institute, Marshfield, Wisconsin; 7 Center for Health Systems Research, Office of Research, Denver Health and Hospital Authority, Denver, Colorado; 8 Department of Epidemiology, Colorado School of Public Health, Aurora

## Abstract

**Importance:**

Uncertainty remains about the longer-term benefits and harms of different opioid management strategies, such as tapering and dose escalation. For instance, opioid tapering could help patients reduce opioid exposure to prevent opioid use disorder, but patients may also seek care elsewhere and engage in nonprescribed opioid use.

**Objective:**

To evaluate the association between opioid dose trajectories observed in practice and patient outcomes.

**Design, Setting, and Participants:**

This retrospective cohort study was conducted in 3 health systems in Colorado and Wisconsin. The study population included patients receiving long-term opioid therapy between 50 and 200 morphine milligram equivalents between August 1, 2014, and July 31, 2017. Follow-up ended on December 31, 2019. Data were analyzed from January 2020 to August 2022.

**Exposures:**

Group-based trajectory modeling identified 5 dosing trajectories over 1 year: 1 decreasing, 1 high-dose increasing, and 3 stable.

**Main Outcomes and Measures:**

Primary outcomes assessed after the trajectory period were 1-year all-cause mortality, incident opioid use disorder, continued opioid therapy at 1 year, and health plan disenrollment. Associations were tested using Cox proportional hazards regression and log-binomial models, adjusting for baseline covariates.

**Results:**

A total of 3913 patients (mean [SD] age, 59.2 [14.4] years; 2767 White non-Hispanic [70.7%]; 2237 female patients [57.2%]) were included in the study. Compared with stable trajectories, the decreasing dose trajectory was negatively associated with opioid use disorder (adjusted hazard ratio [aHR], 0.40; 95% CI, 0.29-0.55) and continued opioid therapy (site 1: adjusted relative risk [aRR], 0.39; 95% CI, 0.34-0.44), but was positively associated with health plan disenrollment (aHR, 1.66; 95% CI, 1.24-2.22). The decreasing trajectory was not associated with mortality (aHR, 1.28; 95% CI, 0.87-1.86). In contrast, the high-dose increasing trajectory was positively associated with mortality (aHR, 2.19; 95% CI, 1.44-3.32) and opioid use disorder (aHR, 1.81; 95% CI, 1.39-2.37) but was not associated with disenrollment (aHR, 0.90; 95% CI, 0.56-1.42) or continued opioid therapy (site 1: aRR, 0.98; 95% CI, 0.94-1.03).

**Conclusions and Relevance:**

In this cohort study, decreasing opioid dose was associated with reduced risk of opioid use disorder and continued opioid therapy but increased risk of disenrollment compared with stable dosing, whereas the high-dose increasing trajectory was associated with an increased risk of mortality and opioid use disorder. These findings can inform opioid management decision-making.

## Key Points

**Question:**

How are 1-year opioid dosing trajectories associated with mortality, opioid use disorder, continued opioid therapy, and health plan disenrollment after the end of the dosing trajectory?

**Findings:**

In this cohort study of 3913 patients, a decreasing opioid dose trajectory was associated with a lower risk of opioid use disorder and continued opioid therapy compared with stable dosing trajectories, but also was associated with an increased risk of disenrollment. Decreasing dose was not associated with mortality in the year after the end of the trajectory period; however, an increasing dose trajectory was associated with an increased risk of mortality and opioid use disorder but had no association with continued opioid therapy or disenrollment.

**Meaning:**

These findings suggest clinicians and patients should carefully weigh the long-term risks and benefits of opioid dose increases and decreases compared with maintaining stable opioid dosing.

## Teaser

This cohort study evaluates the association between opioid dose trajectory and mortality, opioid use disorder, continued opioid therapy, and health plan disenrollment.

## Introduction

Several guidelines support safer opioid management strategies for chronic pain.^1,2,3,4^ For instance, the 2016 Centers for Disease Control and Prevention (CDC) guideline^4^ recommended avoiding opioid dose escalation to 90 or more morphine milligram equivalents (MME), and carefully reassessing risks and benefits when increasing to 50 or more MME. These recommendations were supported by observational studies^5,6,7^ showing that higher doses are associated with increased overdose risk. The guideline also recommends tapering to lower doses or discontinuing opioids, preferably in collaboration with patients using an individualized approach,^3,4^ if patients are not benefiting from opioids or have other overdose risk factors.

Tapering or discontinuing opioids are clinical strategies that may be selected in hopes of preventing sustained opioid exposure and long-term risks of opioid use disorder (OUD), overdose, and death, and may improve function, pain severity, and quality of life.^8^ However, observational studies have identified potential harms of these practices, including increased risks of heroin use,^9^ substance use disorders,^10^ care termination,^11^ overdose or suicide deaths,^12,13^ and emergency department use and hospitalizations for overdose, withdrawal, and mental health crises.^10,14,15^ Additionally, tapering attempts, particularly without shared decision-making, could prompt patients to seek care elsewhere by changing insurance plans or physicians,^11,16^ potentially disrupting care continuity for comorbid health conditions.

Researchers have studied tapering using predefined opioid dosing patterns that occur after periods of stable dosing.^14,15^ However, prior research also suggests that many patients in routine clinical care experience complex and variable dosing patterns over time.^17^ For example, a physician may temporarily increase the dose to treat acute pain, or abruptly discontinue opioids if there is evidence of nonprescribed drug use. This study’s objective was to identify subpopulations of patients with distinct, clinically meaningful trajectory patterns over a 1-year period using group-based trajectory modeling (GBTM).^18,19^ We also aimed to compare baseline characteristics across trajectories and outcomes occurring after the trajectory including 1-year all-cause mortality, incident OUD, continued opioid therapy, disenrollment from the health plan, and overdose.

## Methods

The data-only study was approved by the Kaiser Permanente Colorado institutional review board, with the other sites’ boards ceding oversight to the Kaiser Permanente Colorado board, and was granted a waiver of informed consent in accordance with 45 CFR §46. Study reporting followed the Strengthening the Reporting of Observational Studies in Epidemiology (STROBE) reporting guideline.

### Study Settings

We used data from 3 health systems in 2 states: an integrated insurance provider and health care delivery organization covering Colorado’s urban and suburban regions (site 1), an integrated, safety-net health system serving an urban Colorado region (site 2), and a health care system serving a predominantly rural Wisconsin population (site 3). Data were extracted using a common data model^20^ from their coordinated and quality-controlled secure warehouse research databases; member and patient data were derived from electronic health records (EHRs), health system pharmacies, and external medical or pharmacy insurance claims. Data included demographic information, social history, diagnoses (*International Classification of Diseases*, *Ninth Revision* and *Tenth Revision*), pharmacy dispensations (National Drug Codes), and utilization. To ensure data consistency, we used distributed SAS code and explored differences in covariate frequencies and outcome rates across sites. To ensure complete and consistent capture, vital status and cause-of-death were derived from patient identifiers linked to the National Death Index (NDI)-Plus.^21^

### Study Design and Study Population

This was a retrospective cohort study of patients prescribed long-term opioid therapy between 2014 and 2017. Cohort eligibility started on August 1, 2014, when Colorado Medicaid implemented an opioid tablet limit of 120 per month^22^ and ended on July 31, 2017. Patients needed 3 or more opioid prescriptions dispensed on different dates within 90 days, with gaps of no more than 5 days, medication coverage for at least 80 of the 90 days, and a daily dose of 50 MME or more for at least 30 of the 90 days. The first day of the 3 eligibility dispensations represented the index date. Patients were excluded if they were younger than 18 years on the index date. Due to concerns about limited capture of covariates and medication dispensations, patients were excluded if they had no health plan enrollment (sites 1 and 3) for 12 or more months before the index date or did not meet empanelment criteria^23^ (ie, no primary care visit for 18 months preceding the index date at site 2); lacked pharmacy coverage on the index date; were previously enrolled in hospice or nursing home care; or were in the hospital, skilled nursing facility, or long-term care facility on the index date. Patients with prior skilled nursing stays were eligible since these were generally shorter than hospice or nursing home stays. Finally, persons with MME doses of 200 or greater for 30 or more days during 90 days were excluded to prevent influential outlier dosages. Although opioid prescribing guidelines tend to exclude patients with cancer pain,^1,2,4^ we included patients with previous cancer diagnoses because they may continue to receive opioids after cancer treatment for unrelated pain, thus facing risks commensurate with those experienced by patients without cancer.^24,25^ To establish dosing trajectories during the year after the index date (the trajectory period), we excluded patients who died, were hospitalized, or were institutionalized for 28 or more days, or stopped being enrolled or empaneled.

### Identifying Opioid Dosing Trajectories

Within a large population, GBTM can create meaningful trajectory groups representing patterns of individual change in opioid dose over time, such as maintaining, increasing, or decreasing the dose. Groups are interpreted as latent longitudinal strata, meaning that individuals within a group are assigned a probability of group membership and are assumed to follow the same trajectory over time.^26,27^ Using GBTM, we identified clusters of patients according to their probability of following a similar mean daily MME pattern, calculated in 30-day intervals, over the trajectory period.

**Figure 1.**
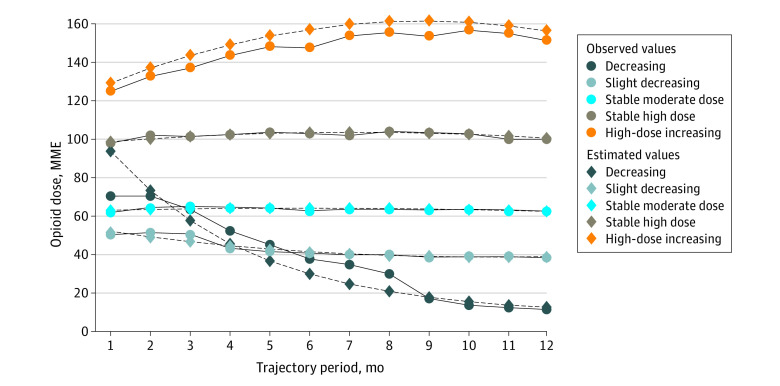
Opioid Dose Trajectories According to Group-Based Trajectory Model Solid lines indicate observed values, and dashed lines indicate estimated values. MME indicates morphine milligram equivalents.

We modeled MMEs over time using a β distribution, which allowed for a diversity of dose patterns for identified groups.^28^ For the β distribution, we scaled the MME values to between 0 and 1 by truncating mean daily values above 400 MME (>99th percentile) during the trajectory period and dividing the values by 400. We assessed model fit with bayesian information criterion and the log Bayes factor approximation and evaluated linear, quadratic, and cubic terms to best fit the data (eTable 1 in the Supplement). We examined models with 1 to 7 trajectory groups; 6-group and 7-group models failed to converge using quadratic terms, and the 6-group model using linear terms had a group that was too small (<5%). Thus, a 5-group model with quadratic terms was considered the optimal model, and all groups had posterior probabilities of 0.95 or greater. We characterized trajectory groups according to our clinical interpretation of the patterns observed in Figure 1: decreasing, slight decreasing, stable moderate dose, stable high dose, and high-dose increasing. We also considered the mean doses in the first and last month of the period, the dose trend, and the proportion who stopped opioids for 1 month or longer during the trajectory. For analysis, we compared the decreasing group and the high-dose increasing group with the other 3 groups (slightly decreasing, stable moderate dose, and stable high dose, together referred to as “stable”) combined.

### Cohort Characteristics

We explored baseline characteristics previously shown to be associated with opioid tapering, discontinuation, dose variability, or overdose mortality.^6,17,29,30,31,32^ Demographic characteristics included age on the index date, sex, and race and ethnicity as reported by patients during visit registration and recorded in the EHR. Race and ethnicity, Medicaid coverage, and the calendar year of the index date were assessed because they could impact management decisions and the outcomes. All available race and ethnicity categories were described, and 2 categories were used for analytic modeling: racial and ethnic minority groups and non-Hispanic White, with 2.2% missing race and ethnicity added to the non-Hispanic White referent group. Medicaid coverage also served as a proxy for low-income status. Cancer, chronic or acute pain,^33^ nonfatal overdose, mental health disorders (eg, depression, anxiety, and bipolar) and tobacco, alcohol, opioid, cannabis, and other (sedative, hypnotic, or anxiolytic; cocaine; other stimulant; hallucinogen; or other psychoactive) substance use disorders were assessed in the year before the index date. Prescribed and dispensed benzodiazepines and stimulants were examined in the 6 months before the index date. Covariates were identified using *International Classification of Diseases *codes, social history fields, National Drug Codes, or other available data fields (eTable 2 in the Supplement) and when present were coded as yes; covariates not present were coded as no. Thus, other than race and ethnicity, there were no missing data.

### Outcomes Following Opioid Dose Trajectories

We examined 4 primary outcomes after the trajectory period ended: all-cause mortality, OUD, continued opioid therapy at 1 year, and disenrollment. Secondary outcomes were fatal and nonfatal overdose from any substance combined and from opioids. Study follow-up ended on December 31, 2019, to avoid COVID-19–related health care disruptions. We limited follow-up for mortality and disenrollment to 1 year because later outcomes were less likely to be associated with the opioid trajectories. We examined all available follow-up time for OUD and overdose because these outcomes could still be associated with dose trajectories after a year. We examined time-to-death regardless of enrollment because NDI data allowed us to identify deaths in any setting. OUD was identified using diagnostic codes in the HER, and overdose was identified using diagnostic codes supplemented with NDI cause-of-death (eTable 2 in the Supplement). Among patients who were enrolled and alive 1 year after the trajectory period ended, we examined any continued opioid therapy. The disenrollment outcome included patients who, for any reason, stopped being enrolled in the health plan (sites 1 and 3) or empaneled (site 2).

### Statistical Analysis

Baseline characteristics were compared using 2-sided χ^2^ tests for categorical variables and means and the Kruskal-Wallis tests for continuous variables. To examine associations of baseline characteristics with the trajectory groups, we used log-binomial models to compare the decreasing and high-dose increasing groups with the combined stable groups (slightly decreasing, stable moderate dose, and stable high dose). We tested for interactions between baseline characteristics and site and included all significant (*P* < .05) interactions in the final model.

We developed regression models to examine associations between group membership and each posttrajectory outcome separately. Disenrollment was examined in a time-to-event analysis starting at the end of the trajectory period, with death as a censoring event. To avoid analyzing dose patterns caused by OUD and overdose, we analyzed time-to-incident OUD, overdoses, and opioid overdoses in patients who did not have each respective diagnosis before the index date. Disenrollment and death were censoring events for OUD and overdose. All models compared the high-dose increasing and decreasing groups with the relatively stable groups. A log-binomial model was fit to examine the continued opioid therapy outcome, whereas Cox proportional hazards regression models were used to examine time-to-each of the other outcomes. Proportional hazards assumptions were confirmed by a review of survival graphs and proportional hazard global tests.^34^ Models were adjusted for all available baseline patient characteristics. We also tested interactions between group membership and site to determine whether trajectory groups had consistent associations with the outcomes across sites.

Sensitivity analyses excluded (1) patients with past-year cancer, to ensure cancer was not a factor in the observed associations, and (2) patients hospitalized during the trajectory period, to ensure limited inpatient opioid dispensing data was not leading to biased results for the mortality outcome. We used all available data for this population-based cohort study. Analyses were completed in SAS Studio statistical software release 3.7 (Enterprise Edition; SAS Institute, Inc) with the add-on procedure Traj by Jones^35^ for GBTM. Data were analyzed from January 2020 to August 2022.

## Results

Across sites, 3913 patients (site 1, 2737 patients; site 2, 601 patients; site 3, 575 patients) prescribed long-term opioid therapy were eligible (eFigure 1 in the Supplement). Differences between patients included in and excluded from the analyses are shown in eTable 3 in the Supplement. At baseline, included patients had a mean (SD) age of 59.2 (14.4) years, 2767 (70.7%) were White non-Hispanic 2237 (57.2%) were female, 815 (20.8%) had Medicaid, and 359 (9.2%) had cancer (Table 1).

**Table 1.** Baseline Demographic and Clinical Characteristics of the Multisite Study Cohort

Characteristic	Patients, No. (%) (N = 3913)
Site	
1	2737 (70.0)
2	601 (15.4)
3	575 (14.7)
Age, mean (SD), y	59.2 (14.4)
Sex	
Male	1676 (42.8)
Female	2237 (57.2)
Race and ethnicity	
American Indian, Native Alaskan, or Hawaiian/Pacific Islander, non-Hispanic	54 (1.4)
Hispanic (Latinx)	633 (16.2)
Non-Hispanic	
Asian	26 (0.7)
Black	293 (7.5)
White	2767 (70.7)
Multiple and other races or ethnicity^a^	53 (1.4)
Missing race and ethnicity	87 (2.2)
Medicaid	815 (20.8)
History of substance use disorders^b^^,^^c^	
Tobacco	940 (24.0)
Alcohol	258 (6.6)
Opioid	205 (5.2)
Cannabis	74 (1.9)
Other substance use disorder	142 (3.6)
Opioid overdose^b^	34 (0.9)
Chronic or acute pain diagnosis^b^	3638 (93.0)
Cancer diagnosis^b^	359 (9.2)
Mental health diagnosis^b^	2210 (56.5)
Benzodiazepine dispensation^d^	1077 (27.5)
Stimulant dispensation^d^	130 (3.3)
Year initiated long-term opioid therapy	
2014	2490 (63.6)
2015	759 (19.4)
2016	470 (12.0)
2017	194 (5.0)

^a^ Other race is a category in the Virtual Data Warehouse which may reflect patient selections at the time of insurance enrollment or visit registration.

^b^ Diagnoses were assessed 1 year before the index date.

^c^ Individuals could have more than 1 substance use disorder. Tobacco was assessed using diagnoses and social history fields. Other substance use disorders including sedative, hypnotic, or anxiolytic; cocaine; other stimulant; hallucinogen; or other psychoactive substance use disorders.

^d^ Dispensations were assessed 6 months before the index date.

### Group-Based Trajectories

Five opioid dosing trajectories were identified, characterized, and described (Figure 1 and Table 2): 1021 patients (26.1%) in the decreasing, 761 patients (19.5%) in the slightly decreasing, 980 patients (25.0%) in the stable moderate dose, 753 patients (19.2%) in the stable high dose, and 398 patients (10.2%) in the high dose increasing trajectory group. Baseline characteristics differed across trajectory groups (eTable 4 in the Supplement). Site adjustment attenuated or eliminated some observed differences (Table 3), but Medicaid coverage, for instance, remained positively associated with the decreasing trajectory (adjusted relative risk [aRR], 1.13; 95% CI, 1.02-1.24) and cancer with the high-dose increasing group (aRR, 2.17; 95% CI, 1.71-2.76). Site interactions were observed (Table 3). For example, racial and ethnic minority group status was only associated with decreasing at site 2 (aRR, 1.26; 95% CI, 1.05-1.51), and site 2 had a higher proportion of patients in the decreasing trajectory compared with sites 1 and 3 (396 of 601 [65.9%] in the site 2 decreasing trajectory compared with 489 of 2737 [17.9%] in site 1, and 136 of 575 [23.7%] in site 3).

**Table 2.** Opioid Dose Trajectories by Site and Dose Characteristics Over the Trajectory Period

Characteristic	Patients, No. (%)					
	Group 1, decreasing	Group 2, slight decreasing	Group 3, stable moderate dose	Group 4, stable high dose	Group 5, high-dose increasing	Total
Total	1021 (26.1)	761 (19.5)	980 (25.0)	753 (19.2)	398 (10.2)	3913 (100)
Site						
1	489 (47.9)	599 (78.7)	760 (77.6)	596 (79.2)	293 (73.6)	2737 (70.0)
2	396 (38.8)	59 (7.8)	63 (6.4)	40 (5.3)	43 (10.8)	601 (15.4)
3	136 (13.3)	103 (13.5)	157 (16.0)	117 (15.5)	62 (15.6)	575 (14.7)
Opioid dose MME, mean (SD)						
Month 1	70.4 (38.0)	50.3 (18.7)	61.9 (15.9)	98.3 (27.4)	124.9 (46.4)	75.3 (37.3)
Month 12	11.4 (26.5)	38.2 (13.9)	62.8 (15.0)	101.2 (25.2)	151.3 (73.5)	61.0 (53.2)
Opioid dose change between trajectory month 1 and 12						
Decrease ≥20%	925 (90.6)	366 (48.1)	161 (16.4)	120 (15.9)	67 (16.8)	1639 (41.9)
Increase ≥20%	29 (2.8)	118 (15.5)	227 (23.2)	190 (25.2)	162 (40.7)	726 (18.6)
Stopped opioids for ≥1 trajectory month	898 (88.0)	0	0	0	13 (3.3)	911 (23.3)

Abbreviation: MME, morphine milligram equivalents.

**Table 3.** Adjusted Associations Between Baseline Characteristics and the Opioid Dose Decreasing Trajectory (Group 1), Increasing Trajectory (Group 5) vs the Relatively Stable Dose Trajectories (Groups 2-4)^a^

Baseline characteristics	Relative risk (95% CI)	
	Decreasing	High-dose increasing
Sex		
Female	1 [Reference]	1 [Reference]
Male	0.91 (0.83-0.99)	1.16 (0.97-1.39)
Medicaid	1.13 (1.02-1.24)	NA
History of use disorder		
Alcohol use disorder	1.00 (0.87-1.15)	0.94 (0.65-1.37)
Opioid use disorder	1.04 (0.86-1.25)	2.00 (1.46-2.75)
Other substance use disorder^b^	0.98 (0.78-1.22)	0.48 (0.24-0.96)
Past-year opioid overdose	1.48 (0.81-2.70)	2.45 (1.36-4.42)
Chronic or acute pain diagnosis	1.41 (1.10-1.80)	0.81 (0.59-1.09)
Mental health diagnosis	0.94 (0.86-1.03)	1.00 (0.82-1.22)
Benzodiazepine dispensation	1.03 (0.94-1.13)	1.08 (0.88-1.32)
Stimulant dispensation	1.19 (0.95-1.49)	1.14 (0.71-1.84)
Age (per 10 y increase)	NA	0.99 (0.99-1.00)
Racial and ethnic minority groups (vs non-Hispanic White)	NA	0.90 (0.71-1.14)
History of cannabis use disorder	NA	0.96 (0.45-2.04)
Tobacco use	NA	1.00 (0.81-1.25)
Cancer in the prior year (vs no history)	NA	2.17 (1.71-2.76)
Year of the index date	NA	0.79 (0.70-0.89)
Interaction between Medicaid and site^c^		
At site 1: Medicaid	NA	0.79 (0.54-1.15)
At site 2: Medicaid	NA	0.97 (0.62-1.52)
At site 3: Medicaid	NA	1.86 (1.22-2.84)
Interaction between age and site, per 10 y^c^		
At site 1: age	0.94 (0.89-1.00)	NA
At site 2: age	1.04 (1.00-1.11)	NA
At site 3: age	0.96 (0.89-1.04)	NA
Interaction between race/ethnicity and site^c^		
At site 1: Racial and ethnic minority groups (vs non-Hispanic White)	0.95 (0.80-1.14)	NA
At site 2: Racial and ethnic minority groups (vs non-Hispanic White)	1.26 (1.05-1.51)	NA
At site 3: Racial and ethnic minority groups (vs non-Hispanic White)	0.52 (0.25-1.10)	NA
Interaction between cannabis use disorder and site^c^		
At site 1: cannabis use disorder	2.24 (1.49-3.37)	NA
At site 2: cannabis use disorder	0.79 (0.54-1.17)	NA
At site 3: cannabis use disorder	1.98 (1.04-3.77)	NA
Interaction between tobacco use and site^c^		
At site 1: tobacco	0.80 (0.64-0.99)	NA
At site 2: tobacco	1.07 (0.87-1.31)	NA
At site 3: tobacco	0.78 (0.59-1.03)	NA
Interaction between cancer diagnosis in the prior year and site^c^		
At site 1: cancer	1.68 (1.40-2.03)	NA
At site 2: cancer	0.99 (0.79-1.24)	NA
At site 3: cancer	0.97 (0.70-1.34)	NA
Interaction between year of index date and site, per year^c^		
At site 1: year	1.46 (1.37-1.55)	NA
At site 2: year	0.76 (0.68-0.84)	NA
At site 3: year	1.28 (1.15-1.42)	NA

Abbreviation: NA, not applicable.

^a^ Log-binomial models.

^b^ Other substance use disorders include sedative, hypnotic, or anxiolytic; cocaine; other stimulant; hallucinogen; or other psychoactive.

^c^ Interactions presented considering site as an effect modifier with other variable effects displayed within site (eg, the relative risk of racial and ethnic minority groups vs non-Hispanic White at Site 1). Significant interactions for decreasing vs stable were found for age (*P* for interaction = .04), racial and ethnic minority groups (*P* for interaction = .02), cannabis use disorder (*P* for interaction < .001), tobacco (*P* for interaction = .01), cancer (*P* for interaction < .001), year of index date (*P* for interaction < .001); for increasing vs stable, Medicaid (*P* for interaction = .02).

### Outcomes After the Trajectory Period

After the trajectory period, 165 cohort members (4.2%) died within a year, the majority from cancer or cardiovascular disease. In the year after the trajectory period, 3499 (89.4%) remained alive and enrolled, and among those, 2531 (72.3%) continued opioid therapy. Over a mean (SD) of 3.0 (1.3) years of follow-up, 401 (11.4%) had an incident OUD and 61 (1.6%) had an overdose (eTable 5 in the Supplement).

**Figure 2.**
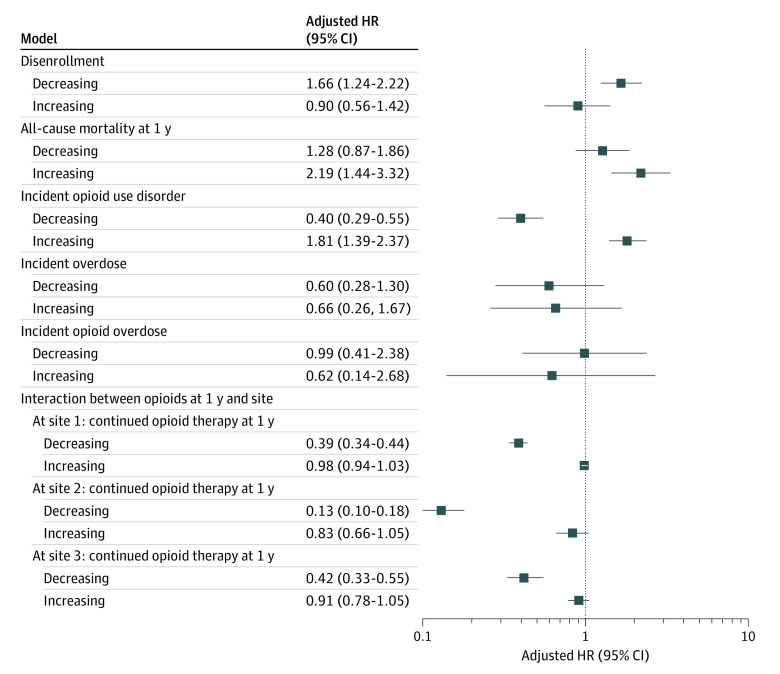
Adjusted Associations Between Increasing and Decreasing Opioid Dose Trajectories and Outcomes Forest plot shows hazard ratios for all outcomes except continued opioid therapy at 1 year, which shows a relative risk.Opioid use disorder and overdose outcomes exclude persons with history of the event; continued opioid therapy at 1 year excluded persons who died or disenrolled before 1 year. Data were adjusted for the site, age, sex, race and ethnicity, smoking status, Medicaid, cancer diagnosis, chronic or acute pain diagnosis, mental health disorder diagnosis, tobacco use/use disorder, alcohol use disorder, opioid use disorder (except for the opioid use disorder outcomes), cannabis use disorder, other substance use disorder, previous opioid overdose (except for the incident overdose and opioid overdose models), previous benzodiazepine dispensation, previous stimulant dispensation, and year of the index date. Opioid use disorder and previous opioid overdose were combined into 1 variable for the continued opioid therapy at 1-year model. HR indicates hazard ratio.

In adjusted analyses, the decreasing trajectory was associated with a reduced OUD incidence (adjusted hazard ratio [aHR], 0.40; 95% CI, 0.29-0.55) but increased disenrollment (aHR 1.66; 95% CI, 1.24-2.22) (Figure 2; see also eTable 5 in the Supplement for event numbers and rates) compared with the relatively stable dose trajectories. Decreasing was not associated with 1-year mortality (aHR, 1.28; 95% CI 0.87-1.86) or overdose (aHR, 0.60; 95% CI, 0.28-1.30). Although decreasing was associated with a reduction in continued opioid therapy at all sites, there was an interaction with site (relative to the stable group at each site, aRR for site 1, 0.39; 95% CI, 0.34-0.44; aRR for site 2, 0.13; 95% CI, 0.10-0.18; aRR for site 3, 0.42; 95% CI, 0.33-0.55; *P *for interaction < .001).

The high-dose increasing trajectory was associated with an increased OUD incidence (aHR, 1.81; 95% CI, 1.39-2.37) and 1-year mortality (aHR, 2.19; 95% CI, 1.44-3.32) compared with the stable dose groups (Figure 2). No significant association was observed between increasing and continued opioid therapy (site 1: aRR, 0.98; 95% CI, 0.94-1.03), disenrollment (aHR 0.90; 95% CI, 0.56-1.42), or overdose (aHR 0.66; 95% CI, 0.26-1.67). Sensitivity analyses (eFigure 2, eTable 6, and eTable 7 in the Supplement) demonstrated similar results, except the association between the high-dose increasing trajectory and mortality was not significant when patients with cancer were excluded (aHR, 1.70; 95% CI, 0.93-3.12).

## Discussion

In this cohort study of opioid dosing trajectories, we identified decreasing, high-dose increasing, and 3 stable trajectories. Although we cannot be certain what clinical strategies or circumstances led to the observed trajectories, the decreasing trajectory appeared consistent with tapering to a lower dose or discontinuing opioids, the high-dose increasing trajectory appeared consistent with dose escalation, and the stable groups appeared consistent with dose maintenance.

After the year-long trajectory period, decreasing was associated with a reduced incidence of OUD and continued opioid therapy compared with stable dosing. However, decreasing was not associated with a significant reduction in 1-year mortality or overdose, and one potential harm was health plan disenrollment. Compared with the stable groups, the high-dose increasing trajectory was associated with an increased risk of death and OUD, but was not associated with continued opioid therapy, disenrollment, or overdose.

Over the trajectory period, more than half of patients maintained opioid dose stability. Compared with stable dosing, however, patients and physicians may be able to further prevent the longer-term development of OUD if they can complete a taper while maintaining health plan enrollment. Although this study cannot explain the association between decreasing dose and disenrollment, abrupt opioid discontinuations, rapid tapers, a lack of mutual agreement between patients and physicians, inadequate ancillary pain management, and inadequate attention to withdrawal symptoms could all contribute to patient dissatisfaction, disengagement, and disenrollment. Loss of insurance or disengagement from ongoing ambulatory care could subsequently have detrimental medical and mental health consequences for patients with chronic pain and comorbid health conditions.^36^ These results can help inform patients, physicians, and health plans or systems about expected outcomes after a dose reduction, but research is needed on the short-term benefits and harms of different opioid management strategies. Further research is needed on how to mitigate any unintended effects of tapers. Future research could also explore health system, insurance, or formulary effects on outcomes. For instance, some observed site differences could have been due to Colorado Medicaid policies limiting opioid tablets dispensed to 120 per 30 days and dose restrictions.^37^

Our findings on the high-dose increasing trajectory support prior research and recommendations that caution about dose escalation,^4,25,38^ but should be interpreted in context. Despite accounting for measurable confounders, the positive association between increasing and mortality could be due to unmeasured factors related to the underlying risk of death among people selected for dose increases or the reasons dose increases were initiated, such as unremitting pain or poor health status.

### Limitations

This study has limitations. Within-group heterogeneity is obscured using GBTM. For instance, the shape of the decreasing trajectory in Figure 1 does not necessarily reflect the pace at which all patients in that group reduce their doses. A minimum period of event-free survival is required to assess trajectories, underestimating adverse event rates. Thus, the association between trajectories and outcomes occurring during the trajectory period, rather than after the trajectory, could not be evaluated with this approach. All diagnoses were recorded in routine clinical care, which could have led to inaccuracies. Data on education, income, and other potential confounders were unavailable. Although we excluded prior OUD or overdose to minimize reverse causation in the analysis of those outcomes, we cannot exclude the possibility that a nonfatal overdose during the trajectory period influenced opioid management and, therefore, group membership.

GBTM has been applied to data across many Department of Veterans Affairs clinics,^19^ but has not been extensively applied to multisite studies from different types of health systems; thus, we explored site interactions in all models. Low numbers from 2 sites precluded robust site-specific estimates and larger sample size is needed to definitively test the association between decreasing and overdose, given findings reported by Agnoli and colleagues.^14^ Additionally, overdoses not leading to a hospital visit or death could not be identified using our methods. Despite these limitations, the health systems studied serve 2 states with distinct prescribing policies, care delivery systems, and insurance products, enhancing generalizability.

## Conclusions

Our findings suggest that physicians and patients should be informed of the longer-term benefits and risks of opioid dose increases and decreases compared with maintaining dose stability. If dose decreases are indicated, clinicians should consider how to mitigate the risk of disenrollment. If dose increases are indicated among patients on high doses, patients should be monitored for the development of opioid use disorder. Research on short-term effects of opioid dose changes is also needed.

## Notes

Supplement. **eTable 1.** Model Fit Criteria Used to Select the Number of Trajectory Groups **eTable 2.** *International Classification of Diseases Codes* (*ICD*-*9/10*) and Other Codes Used to Identify Outcomes and Covariates **eFigure 1.** Flow Diagram for the Study Cohort From 3 Health System Sites **eTable 3.** Baseline Characteristics of Eligible Patients Included in the Analyses Compared With Eligible Patients Not Included in the Analyses **eTable 4.** Baseline Demographic and Clinical Characteristics by Each of the 5 Trajectory Groups **eTable 5.** Study Outcomes in the Overall Cohort and in Each of the 5 Trajectory Groups **eFigure 2.** Excluding Patients with Cancer at Baseline, Adjusted Associations Between Decreasing and Increasing Opioid Dose Trajectory vs Stable Groups and Outcomes (Sensitivity Analysis) **eTable 6.** Number of Deaths in the Study Cohort Used for the Primary Analysis Compared With the Cohort Excluding Patients Hospitalized During the Trajectory Period (Sensitivity Analysis), by Trajectory Group **eTable 7.** Adjusted Associations Between Decreasing and Increasing Opioid Dose Trajectory vs Stable Groups and 1-year Mortality in the Study Population Used for the Primary Analysis and After Excluding Individuals Hospitalized During the Trajectory Period (Sensitivity Analysis)
