# Supplementary material for: Opioid Dose Trajectories and Associations With Mortality, Opioid Use Disorder, Continued Opioid Therapy, and Health Plan Disenrollment
Source: JAMA Netw Open. 2022 Oct 5;5(10):e2234671. doi: 10.1001/jamanetworkopen.2022.34671 (PMC9535531; doi:10.1001/jamanetworkopen.2022.34671)
Supplement: Supplement. — eTable 1. Model Fit Criteria Used to Select the Number of Trajectory Groups eTable 2. International Classification of Diseases Codes (ICD-9/10) and Other Codes Used to Identify Outcomes and Covariates eFigure 1. Flow Diagram for the Study Cohort From 3 Health System Sites eTable 3. Baseline Characteristics of Eligible Patients Included in the Analyses Compared With Eligible Patients Not Included in the Analyses eTable 4. Baseline Demographic and Clinical Characteristics by Each of the 5 Trajectory Groups eTable 5. Study Outcomes in the Overall Cohort and in Each of the 5 Trajectory Groups eFigure 2. Excluding Patients with Cancer at Baseline, Adjusted Associations Between Decreasing and Increasing Opioid Dose Trajectory vs Stable Groups and Outcomes (Sensitivity Analysis) eTable 6. Number of Deaths in the Study Cohort Used for the Primary Analysis Compared With the Cohort Excluding Patients Hospitalized During the Trajectory Period (Sensitivity Analysis), by Trajectory Group eTable 7. Adjusted Associations Between Decreasing and Increasing Opioid Dose Trajectory vs Stable Groups and 1-year Mortality in the Study Population Used for the Primary Analysis and After Excluding Individuals Hospitalized During the Trajectory Period (Sensitivity Analysis) [file jamanetwopen-e2234671-s001.pdf]

Supplemental Online Content

Binswanger IA, Shetterly SM, Xu S, et al. Opioid dose trajectories and associations with mortality, opioid use disorder, continued opioid therapy, and health plan disenrollment. *JAMA Netw Open*. 2022;5(10):e2234671. doi:10.1001/jamanetworkopen.2022.34671

eTable 1. Model Fit Criteria Used to Select the Number of Trajectory Groups

eTable 2. *International Classification of Diseases Codes* (*ICD-9/10*) and Other Codes Used to Identify Outcomes and Covariates

eFigure 1. Flow Diagram for the Study Cohort From 3 Health System Sites

eTable 3. Baseline Characteristics of Eligible Patients Included in the Analyses Compared with Eligible Patients Not Included in the Analyses

eTable 4. Baseline Demographic and Clinical Characteristics by Each of the 5 Trajectory Groups

eTable 5. Study Outcomes in the Overall Cohort and in Each of the 5 Trajectory Groups

eFigure 2. Excluding Patients With Cancer at Baseline, Adjusted Associations Between Decreasing and Increasing Opioid Dose Trajectory vs Stable Groups and Outcomes (Sensitivity Analysis)

eTable 6. Number of Deaths in the Study Cohort Used for the Primary Analysis Compared with the Cohort Excluding Patients Hospitalized During the Trajectory Period (Sensitivity Analysis), by Trajectory Group

eTable 7. Adjusted Associations Between Decreasing and Increasing Opioid Dose Trajectory vs Stable Groups and 1-year Mortality in the Study Population Used for the Primary Analysis and After Excluding Individuals Hospitalized During the Trajectory Period (Sensitivity Analysis)

This supplemental material has been provided by the authors to give readers additional information about their work.

© 2022 Binswanger IA et al. *JAMA Network Open.*

eTable 1. Model Fit Criteria Used to Select the Number of Trajectory Groups[a]

Number of groups	Bayesian Information Criteria (BIC)	Estimate of log Bayes factor[b]	Smallest group percentage[c]	Optimal model
1	40149.3		100.0	
2	58465.0	36631.4	38.7	
3	69055.1	21180.2	27.6	
4	71898.9	5687.6	11.6	
5	75040.3	6282.8	10.2	***[d]
6	77596.3	5112.0	4.6	

[a] All models were fitted using beta distribution and Proc Traj procedure in SAS. The estimate of log Bayes factor and the smallest group percentage were used to assess the model fit.

[b] The log Bayes Factor is approximately equal to 2 times the difference in the BIC values between the 2 models being compared. The BIC value of the simpler model (i.e., with 1 less trajectory group) is subtracted from the BIC value of more complex model, and the resulting value is multiplied by 2. An estimated log Bayes factor larger than 10 is considered as strong evidence in favor of the more complex model.

[c] We required that no group have less than 5% of the study population contributing to it.

[d] Models with 1 to 7 groups were initially evaluated with quadratic terms. Models with 6 or more groups and all quadratic terms failed to converge. The 6-group model was then evaluated with all linear terms; while the log Bayes Factor indicated that it had a better fit than the 5-group model with all quadratic terms, one of the 6 groups had less than 5% of the population contributing to it. Thus, the model with 5 groups was considered optimal. Models with cubic and linear terms in the 5-group model were then explored and the 5-group model with all quadratic terms remained the model with the best fit.

© 2022 Binswanger IA et al. *JAMA Network Open.*

eTable 2. ***International Classification of Diseases Codes (ICD-9/10)*** **and Other Codes Used to Identify Outcomes and Covariates**

Diagnoses	ICD-9 codes	ICD-10 codes
Drug overdoses (non-fatal)	960.x-976.x, 977.0-977.4, 978.x, 979.x, E850.x-E858.x, E950.0-E950.3, E980.0-E980.3	T36.x-T49.x (excluding underdosing and adverse effect codes), T50.0-T50.8
Drug overdoses (fatal)		Underlying cause of death: X40-X44, X60-X64, X85, Y10-Y14
Opioid overdoses (non-fatal)	965.00, 965.01, 965.02, 965.09, E850.0, E850.1, E850.2	T40.0X1-T40.0X4, T40.1X1-T40.1X4, T40.2X1-T40.2X4, T40.3X1-T40.3X4, T40.4X1-T40.4X4
Opioid overdoses (fatal)		Underlying cause of death: X40–X44, X60–X64, X85, Y10–Y14, and a contributing cause of death code indicating opioid involvement (T40.0, T40.1, T40.2, T40.3, T40.4)
Opioid use disorder	304.0, 304.7, 305.5	F11.x
Other substance use disorder (sedative-hypnotic/anxiolytic; cocaine; other stimulant; hallucinogen; or other psychoactive substance)	292.x, 304.1, 304.2, 304.4-304.6, 304.8, 304.9, 305.3, 305.4, 305.6-305.9	F13.x-F16.x, F18.x, F19.x
Cannabis use disorder	304.3, 305.2	F12.x
Alcohol use disorder	291.x, 303.x, 305.0	F10.x
Tobacco use and use disorder[a]	305.1	F17.x, Z72.0
Chronic or acute pain diagnosis	274.x, 307.8, 337.2, 338.0, 338.2, 338.4, 339.x, 346.x, 350.x, 353.0, 353.6, 354.x, 355.71, 355.9, 710.x-717.x, 718.0, 718.1, 719.x-722.x, 723.0-723.3, 724.x-726.x, 727.0-727.4, 728.x, 729.0-729.2, 729.5, 729.7, 780.96, 784.0	B02.22, D48.1, F45.4, G43.x, G44.x, G50.x, G54.0, G54.6, , G56.x, G57.7, G58.7, G58.9, G89.0, G89.2, G89.4, G90.5, M00.x-M02.x, M05.x-M08.x, M10.x, M11.x, M12.0, M12.1, M12.5, M12.8, M12.9, M14.x-M19.x, M1A.x, M20.1, M21.61, M21.62, M22.4, M23.x, M24.0, M24.1, M24.20, M25.x, M31.5, M32.x-M36.x, M43.2, M43.8, M45.x, M46.0, M46.1, M46.4, M46.5, M46.8, M46.9, M47.x, M48.0, M48.1-M48.3, M48.8, M48.9, M49.8, M50.x, M51.x, M53.0-M53.3, M53.8, M53.9, M54.03-M54.09, M54.1-M54.6, M54.81, M54.89, M54.9, M60.x-M62.x, M65.x, M67.3, M67.4, M70.0-M70.7, M71.1, M71.3, M71.5, M72.x, M75.x, M76.x, M77.x, M79.0-M79.2, M79.6, M79.7, M79.A, M96.1, R51.X, R52.X, S12.x-S14.x, S22.x-S24.x, S32.x-S34.x, S83.x
Cancer	140.x-172.x, 174.x-208.x, 238.6	C00.x-C26.x, C30.x-C34.x, C37.x-C41.x, C43.x, C45.x-C58.x, C60.x-C85.x, C88.x, C90.x-C97.x
Mental health disorder diagnosis	Identified from the Mental Health Research Network https://github.com/MHResearchNetwork/Diagnosis-Codes/blob/master/mhrn_dx_codes_2020.zip Included diagnosis codes for depression, anxiety, bipolar, schizophrenia, affective disorders, eating disorders, post-traumatic stress disorder, and suicide ideation	
Benzodiazepines	Assessed using National Drug Codes	
Stimulants	Assessed using National Drug Codes	

[a] Also assessed using social history table.

© 2022 Binswanger IA et al. *JAMA Network Open.*

eFigure 1. Flow Diagram for the Study Cohort From 3 Health System Sites

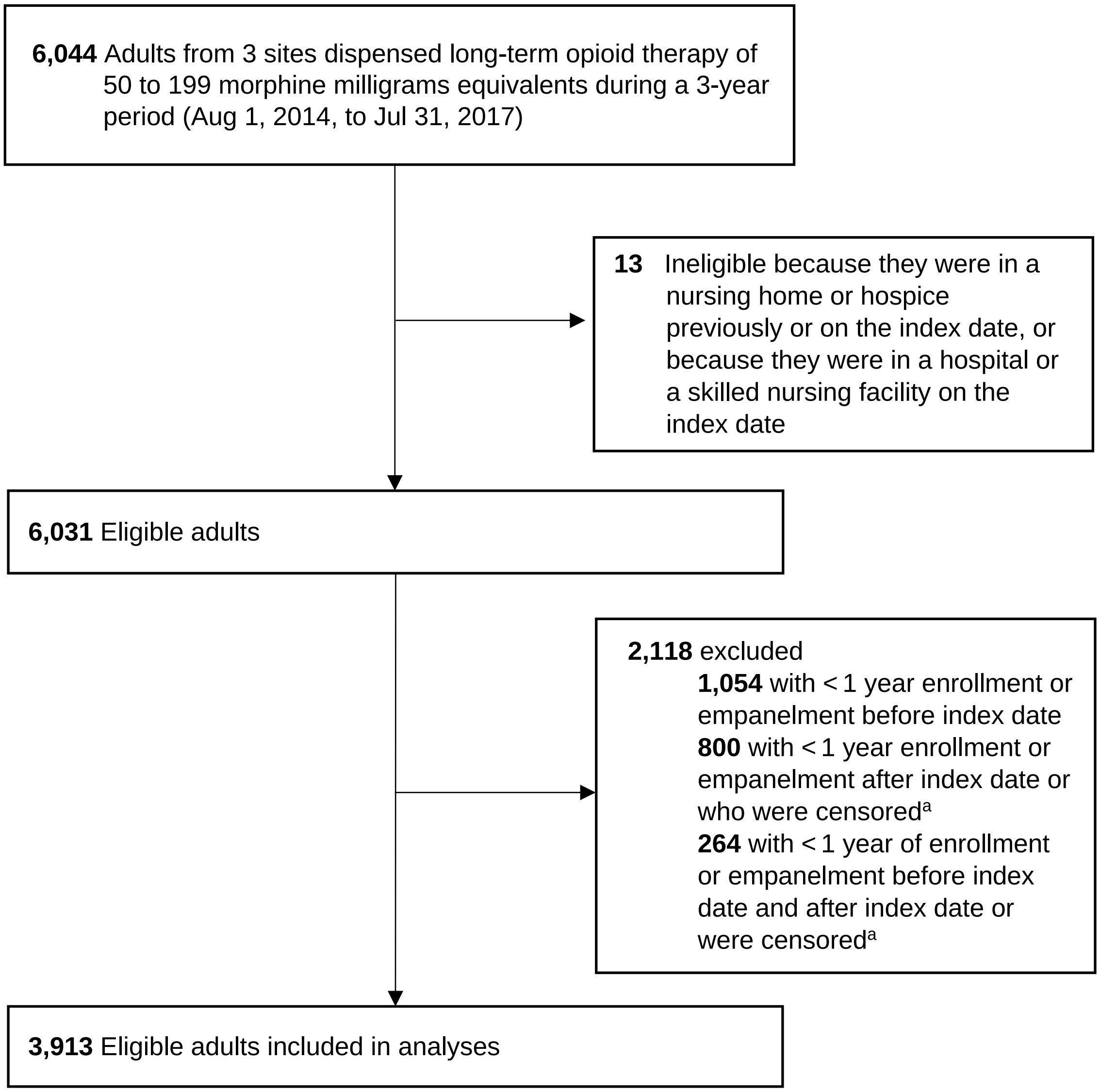

[a] Patients were excluded from the analyses if they did not have a complete year in which trajectories were established. In addition to disenrollment, reasons they could not establish a trajectory were nursing home or other institutional stay or hospitalization of ≥ 28 days and death. These categories were not mutually exclusive and included 337 deaths during the trajectory period.

© 2022 Binswanger IA et al. *JAMA Network Open.*

eTable 3. Baseline Characteristics of Eligible Patients Included in the Analyses Compared with Eligible Patients Not Included in the Analyses

**Characteristic**	**Included in the analyses (n=3,913)**	**Not included in the analyses (n=2118)**	***P* value**
**Site, n (%)**			< 0.001
1	2,737 (70.0)	1,365 (64.5)	
2	601 (15.4)	258 (12.2)	
3	575 (14.7)	495 (23.4)	
**Age, mean y (SD)**	59.2 (14.4)	58.0 (15.5)	0.004
**Sex, n (%)**			
Men	1,676 (42.8)	883 (41.7)	0.39
Women	2,237 (57.2)	1,235 (58.3)	
**Race and Ethnicity, n (%)**			0.06
American Indian, Native Alaskan, or Hawaiian/Pacific Islander, non-Hispanic	54 (1.4)	29 (1.4)	
Asian, non-Hispanic	26 (0.7)	8 (0.4)	
Black, non-Hispanic	293 (7.5)	166 (7.8)	
Hispanic (Latinx)	633 (16.2)	309 (14.6)	
White, non-Hispanic	2,767 (70.7)	1,502 (70.9)	
Multiple and other race or ethnicity	53 (1.4)	31 (1.5)	
Missing race and ethnicity	87 (2.2)	73 (3.5)	
**Medicaid, n (%)**	815 (20.8)	699 (33.1)	< 0.001
**Year of the index date, n (%)**			< 0.001
2014	2,490 (63.6)	837 (39.5)	
2015	759 (19.4)	576 (27.2)	
2016	470 (12.0)	534 (25.2)	
2017	194 (5.0)	171 (8.1)	

© 2022 Binswanger IA et al. *JAMA Network Open.*

eTable 4. Baseline Demographic and Clinical Characteristics by Each of the 5 Trajectory Groups

**Baseline characteristic[a, b]**	**Group 1, decreasing n=1,021 (26.1%)**	**Group 2, slight decreasing n=761 (19.5%)**	**Group 3, stable moderate dose n=980 (25.0%)**	**Group 4, stable high dose n=753 (19.2%)**	**Group 5, high-dose increasing n=398 (10.2%)**
**Age,[b] mean years (SD)**	57.3 (14.1)	60.3 (15.0)	60.7 (14.7)	59.1 (14.1)	58.3 (13.3)
**Male, n (%)**	428 (41.9)	315 (41.4)	413 (42.1)	329 (43.7)	191 (48.0)
**Ethnicity / Race, n (%)[a]**					
American Indian, Native Alaskan, or Hawaiian/Pacific Islander, non-Hispanic	7 (0.7)	9 (1.2)	16 (1.6)	13 (1.7)	9 (2.3)
Asian, non-Hispanic	7 (0.7)	4 (0.5)	8 (0.8)	3 (0.4)	4 (1.0)
Black, non-Hispanic	116 (11.4)	52 (6.8)	62 (6.3)	39 (5.2)	24 (6.0)
Hispanic (Latinx)	256 (25.1)	122 (16.0)	124 (12.7)	92 (12.2)	39 (9.8)
White, non-Hispanic	604 (59.2)	549 (72.1)	733 (74.8)	577 (76.6)	304 (76.4)
Multiple and other race or ethnicity	13 (1.3)	7 (0.9)	11 (1.1)	11 (1.5)	11 (2.8)
Missing race and ethnicity	18 (1.8)	18 (2.4)	26 (2.7)	18 (2.4)	7 (1.8)
**Medicaid, n (%)[a]**	322 (31.5)	124 (16.3)	159 (16.2)	136 (18.1)	74 (18.6)
**History of substance use disorders, n (%)**					
Tobacco[a]	290 (28.4)	156 (20.5)	225 (23)	172 (22.8)	97 (24.4)
Alcohol	85 (8.3)	42 (5.5)	58 (5.9)	47 (6.2)	26 (6.5)
Opioid[a]	50 (4.9)	23 (3.0)	46 (4.7)	51 (6.8)	35 (8.8)
Cannabis[a]	31 (3.0)	9 (1.2)	13 (1.3)	15 (2.0)	6 (1.5)
Other substance use disorder[a]	44 (4.3)	17 (2.2)	34 (3.5)	39 (5.2)	8 (2.0)
**Past year opioid overdose, n (%)[a]**	14 (1.4)	1 (0.1)	9 (0.9)	3 (0.4)	7 (1.8)
**Chronic or acute pain diagnosis, n (%)[a]**	976 (95.6)	694 (91.2)	901 (91.9)	707 (93.9)	360 (90.5)
**Cancer diagnosis, n (%)[a]**	114 (11.2)	58 (7.6)	75 (7.7)	51 (6.8)	61 (15.3)
**Mental health diagnosis, n (%)[a]**	563 (55.1)	414 (54.4)	538 (54.9)	465 (61.8)	230 (57.8)
**Benzodiazepine dispensation, n (%)**	282 (27.6)	200 (26.3)	256 (26.1)	222 (29.5)	117 (29.4)
**Stimulant dispensation, n (%)**	38 (3.7)	23 (3.0)	30 (3.1)	24 (3.2)	15 (3.8)
**Year of index date, n (%)[a]**					
2014	572 (56.0)	385 (50.6)	661 (67.5)	576 (76.5)	296 (74.4)
2015	216 (21.2)	193 (25.4)	190 (19.4)	106 (14.1)	54 (13.6)
2016	170 (16.7)	129 (17.0)	90 (9.2)	45 (6.0)	36 (9.1)
2017	63 (6.2)	54 (7.1)	39 (4.0)	26 (3.5)	12 (3.0)

[a] Chi-square p value < 0.05.
[b] Kruskal-Wallis p value < 0.05.

© 2022 Binswanger IA et al. *JAMA Network Open.*

eTable 5. Study Outcomes in the Overall Cohort and in Each of the 5 Trajectory Groups

Study outcome	Eligible population[a]	No. of events (%)	Rate (n/100 person-years)	Group 1, decreasing, No. of events/No. eligible (%)	Group 2, slight decreasing, No. of events/No. eligible (%)	Group 3, stable moderate dose, No. of events/No. eligible (%)	Group 4, stable high dose, No. of events/No. eligible (%)	Group 5, high-dose increasing, No. of events/No. eligible (%)
Disenrollment/Dis-empanelment at 1 year	3,913	249 (6.4)	6.7	81/1,021 (7.9)	44/761 (5.8)	60/980 (6.1)	43/753 (5.7)	21/398 (5.3)
One-year all-cause mortality	3,913	165 (4.2)	4.3	45/1,021 (4.4)	24/761 (3.2)	35/980 (3.6)	29/753 (3.9)	32/398 (8.0)
Incident opioid use disorder[b]	3,508	401 (11.4)	4.1	52/899 (5.8)	74/712 (10.4)	111/904 (12.3)	94/661 (14.2)	70/332 (21.1)
Incident overdose[c]	3,820	61 (1.6)	0.5	9/983 (0.9)	11/753 (1.5)	19/961 (2.0)	17/738 (2.3)	5/385 (1.3)
Incident opioid overdose[d]	3,873	33 (0.9)	0.3	9/1,005 (0.9)	2/758 (0.3)	8/971 (0.8)	12/748 (1.6)	2/391 (0.5)
Continued opioid therapy at 1 year	3,499	2,531 (72.3)	72.3	223/895 (24.9)	587/693 (84.7)	804/885 (90.9)	626/681 (91.9)	291/345 (84.4)

[a] Incident opioid use disorder and overdose outcomes each exclude persons with a prior history of the respective event; thus, numbers are lower than 3913. Continued opioid therapy at 1 year excludes persons who died or disenrolled before 1 year; thus, numbers are lower than 3913.

[b] Estimated over a median of 1,062 days of follow-up.

[c] Estimated over a median of 1,186 days of follow-up.

[d] Estimated over a median of 1,196 days of follow-up.

© 2022 Binswanger IA et al. *JAMA Network Open.*

eFigure 2. Excluding Patients With Cancer at Baseline, Adjusted[a,b,c] Associations Between Decreasing and Increasing Opioid Dose Trajectories vs Stable Groups and Outcomes (Sensitivity Analysis)

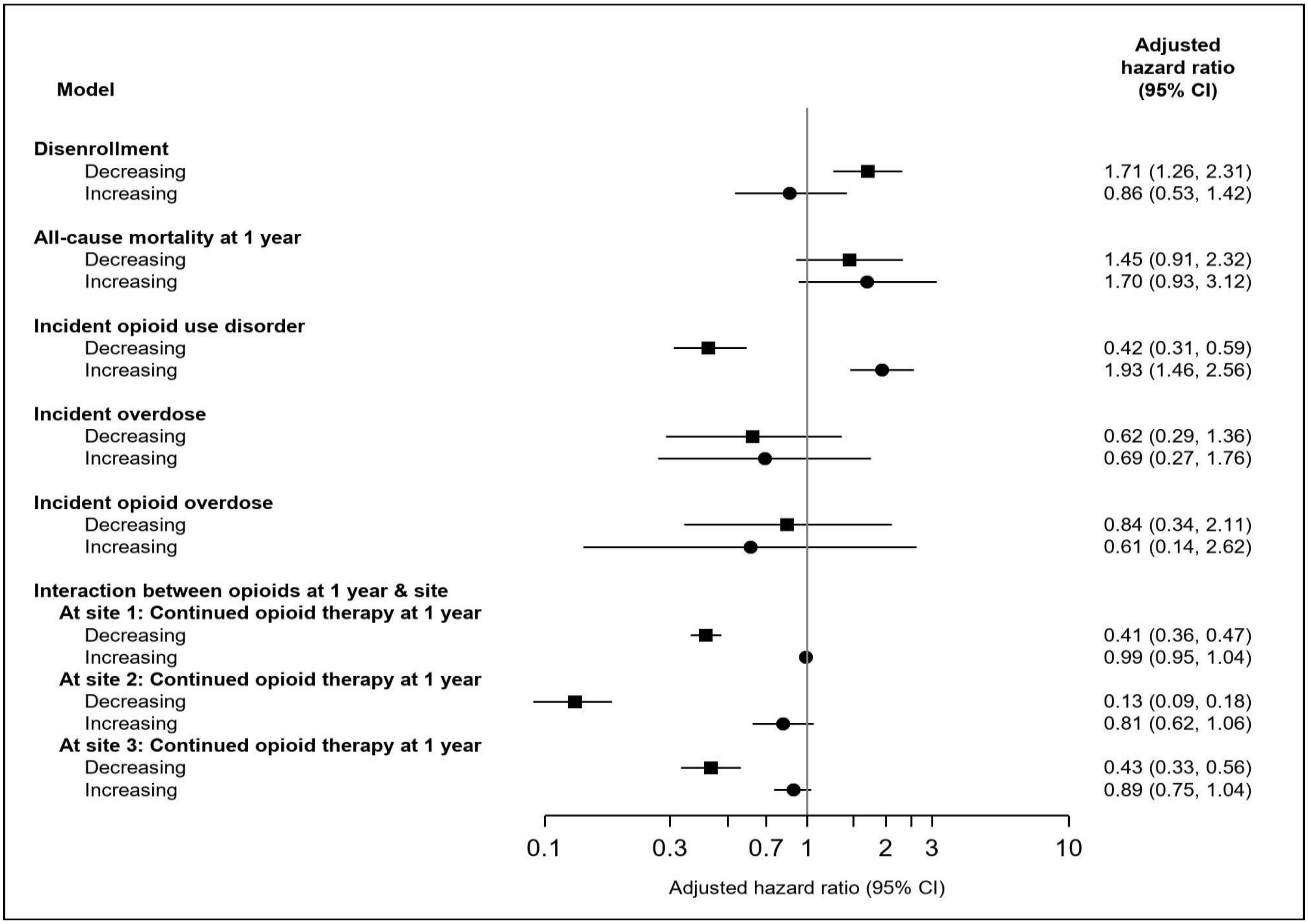

[a] Relative risk for continued opioid therapy at 1 year.

[b] Opioid use disorder and overdose outcomes each exclude persons with prior history of the respective event; Dispensed opioids at 1 year excluded persons who died or disenrolled before 1 year.

[c] Adjusted for the site, age, sex, race/ethnicity, smoking status, Medicaid, chronic or acute pain diagnosis, mental health disorder diagnosis, tobacco use/use disorder, alcohol use disorder, opioid use disorder (except for the opioid use disorder outcomes), cannabis use disorder, other substance use disorder, previous opioid overdose (except for the incident overdose and opioid overdose models), previous benzodiazepine dispensation, previous stimulant dispensation, and year of index date. Opioid use disorder and previous opioid overdose were combined into one variable for the continued opioid therapy at 1 year model.

© 2022 Binswanger IA et al. *JAMA Network Open.*

eTable 6. Number of Deaths in the Study Cohort Used for the Primary Analysis Compared With the Cohort Excluding Patients Hospitalized[a] During the Trajectory Period (Sensitivity Analysis), by Trajectory Group

Trajectory groups	Primary analysis		Sensitivity analysis	
	Deaths (n=165)	Number of individuals (N=3913)	Deaths (n=109)	Number of individuals (N=3198)
Decreasing, n (%)	45 (27.3)	1021 (26.1)	31 (28.4)	785 (24.6)
Stable, n (%)	88 (53.3)	2494 (63.7)	54 (49.5)	2091 (65.4)
Increasing, n (%)	32 (19.4)	398 (10.2)	24 (22.0)	322 (10.1)

[a] Patients hospitalized for ≥ 28 days were not included in the primary analysis

© 2022 Binswanger IA et al. *JAMA Network Open.*

eTable 7. Adjusted Associations[a] Between Decreasing and Increasing Opioid Dose Trajectory vs Stable Groups and 1-year Mortality in the Study Population Used for the Primary Analysis and After Excluding Individuals Hospitalized During the Trajectory Period (Sensitivity Analysis)

	Adjusted hazard ratio (95% CI)	
	Primary analysis	Sensitivity analysis
Decreasing vs stable groups	1.28 (0.87, 1.86)	1.86 (1.17, 2.97)
Increasing vs stable groups	2.19 (1.44, 3.32)	2.80 (1.70, 4.60)

[a] Adjusted for site, age, sex, race/ethnicity, smoking status, Medicaid, cancer diagnosis, chronic or acute pain diagnosis, mental health disorder diagnosis, tobacco use/use disorder, alcohol use disorder, opioid use disorder, cannabis use disorder, other substance use disorder, previous opioid overdose, previous benzodiazepine dispensation, previous stimulant dispensation, and year of index date.

© 2022 Binswanger IA et al. *JAMA Network Open.*
